# Bacterial reduction in sealed caries lesions is strain- and material-specific

**DOI:** 10.1038/s41598-018-21842-8

**Published:** 2018-02-28

**Authors:** Teresa Marggraf, Petra Ganas, Sebastian Paris, Falk Schwendicke

**Affiliations:** Department of Operative and Preventive Dentistry, Charité - Universitätsmedizin Berlin, Berlin, Germany

## Abstract

Sealing can arrest caries lesions. We aimed to evaluate if sealing effects and kinetics are bacterial-strain and sealing-material specific. Human dentin discs were mounted in a dual-chamber device. Caries lesions were induced chemically and contaminated with either *Lactobacillus rhamnosus* (LR) or *Streptococcus sobrinus* (SS). For (1) kinetics assessment, the initial bacterial load and the sealing period were varied, and lesions sealed using a self-etch adhesive and composite. For (2) comparing materials, six sealing protocols (#1-#6) were evaluated: 1# Self-etch adhesive plus composite placed without a liner, or #2 calcium hydroxide, or #3 mineral trioxide aggregate, or #4 Biodentine liners; #5 antibacterial adhesive plus composite; #6 glass ionomer cement. Pulpal fluid flow was simulated during sealing. The outcome was the number of surviving bacteria (CFU) per g dentin. For LR, bacterial survival increased significantly with increasing initial bacterial load and decreased with longer sealing periods. The relative reduction followed a first-order kinetics. More LR survived under calcium hydroxide or MTA than other materials (p < 0.001). For SS, nearly no bacteria survived sealing regardless of sealing period, initial bacterial load or sealing material. In conclusion, sealing effects and kinetics were strain- and material-specific.

## Introduction

For deep carious lesions, the traditional non-selective (‘complete’) carious tissue removal is not recommended any longer^1^. Instead, selective (‘incomplete’) removal, i.e. leaving soft dentin in proximity to the pulp, is thought to reduce the risk of pulp exposure and postoperative pulp complications and thus retain teeth for longer^2,3^. Alternatively, stepwise removal can be performed, i.e. sealing carious tissue beneath a temporary restoration and re-entering the cavity and removing the remaining soft tissue after a certain time^4^. In both approaches, carious tissues are sealed and bacteria remain beneath the placed restoration.

Theoretically, the placed restoration impedes dietary carbohydrate supply of sealed bacteria, which should lead to bacterial inactivation and lesion arrest^5,6^. However, a number of questions remain: First, the survival kinetics of sealed bacteria is unclear, but clinically relevant: For example, in stepwise tissue removal, different sealing times can be chosen by the dentist, with longer sealing periods possibly increasing the chance of killing most bacteria (and also inducing reactionary dentin), but also reducing compliance and leading to possible failure of the temporary restoration^7^. Second, survival of sealed bacteria might depend on the number of sealed bacteria, with large numbers of bacteria (as a result of leaving soft or very soft instead of leathery or firm dentin, for example) being more difficult to inactivate. In this case, providing antibacterial cavity treatments or using antibacterial restorative strategies could be relevant. Third, in deep lesions, it has been shown that sealed bacteria may be supplied with pulpal fluids, which contain glycoproteins and amino acids^8^. Some bacteria can metabolize such substrates and thus survive sealing-induced starvation^9^. Understanding such stress response and the associated ecologic selection processes could help to tailor clinical interventions^10^.

Clinical studies can theoretically yield such understanding. However, as growing evidence finds any cavity re-entry possibly detrimental to pulpal health^7,11^, repeated sampling of sealed bacteria to investigate their inactivation or metabolic activity is debatable. Moreover, a number of clinical factors, like residual dentin thickness, pulp fluid composition and pressure as well as the bacterial composition of sealed lesions are highly variable between patients and teeth, which makes both analysis and interpretation of clinical findings challenging. Yielding sufficient biomasses for more advanced transcriptomic or metabolomics analyses is additionally difficult. Last, clinical studies are unsuitable for assessing interventions which have not been tested otherwise so far (like experimental materials). In conclusion, a preclinical testing system is needed which allows to evaluate the bacterial reaction to sealing. Such system should incorporate the simulation of pulpal fluid flow, as this might be decisive for any sealing effects in a clinical setting. Using such preclinical testing system would also allow to standardize carious lesions (with regards to bacterial numbers and strains), the residual dentin thickness, and the pulpal fluid composition and pressure.

In the present study, we employed such a system, aiming to evaluate the survival kinetics of two sealed bacterial strains, namely *Lactobacillus rhamnosus* (LR) and *Streptococcus sobrinus* (SS). Our hypothesis was that longer sealing periods are associated with significantly lower numbers of remaining bacteria, and that the number of sealed bacteria is significantly associated with the number of eventually surviving bacteria. We hypothesized that these effects would be found for both strains, i.e. not strain-specific. Moreover, we compared the effects of different restorative strategies on sealed bacteria, hypothesizing that different materials would yield significantly different numbers of surviving sealed bacteria.

**Figure 1:**
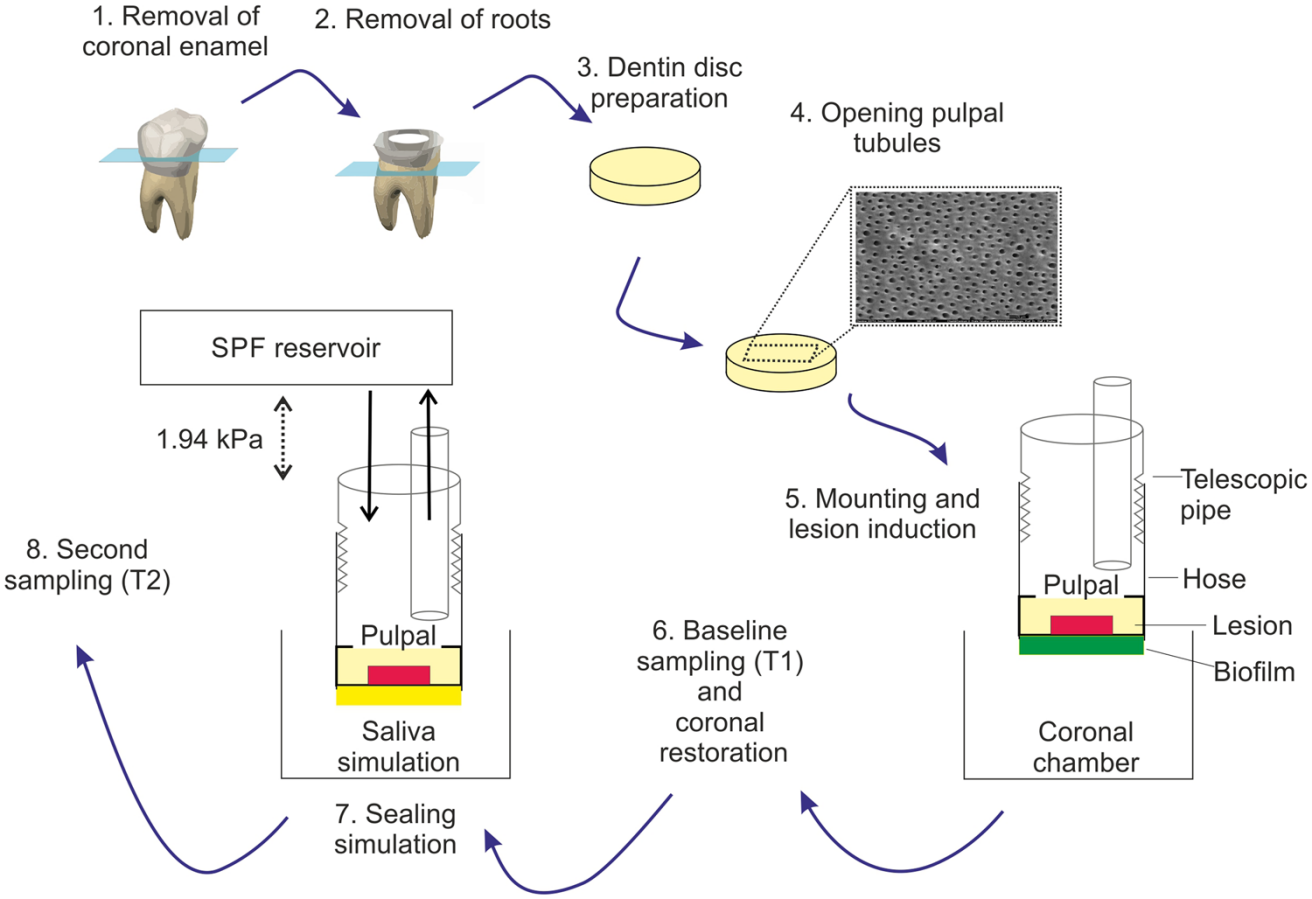
Experimental set-up. After preparing the dentin discs by cutting-out dentin sections from human third molars (1–3), and opening the dentinal tubules from pulpal using EDTA (4, see exemplary SEM image), caries lesions (red) induced coronally using biofilms (green) after mounting (5), as shown (for *L. rhamnosus*) or before mounting (for *S. sobrinus*). The system consisted of a coronal (mouth) chamber (where the biofilm was cultured) and a pulpal chamber (see main text). After sampling dentin for the determination of the initial bacterial load (T1), samples were sealed using restorative material (yellow) and submitted to the sealing simulation period (6–7), with artificial saliva being provided coronally and simulated pulpal fluid (SPF) circulation (under a pressure of 1.94 kPa) pulpally (indicated by arrows). At the conclusion of the experiment (T2), bacteria were re-sampled (8).

In accordance with these aims, the study first assessed the survival kinetics of two sealed bacterial strains found in dentin carious lesions, LR and SS. These served to exemplify possible strain-specific sealing effects, while clinically, a more complex and diverse bacterial flora would be sealed. In the second part of the study, we compared different restorative materials, as might be used by dentists, for their effects on bacteria survival. Both experiments were performed using a novel dual-chamber device which allows to assess survival of bacteria sealed under dental restorations, as it incorporates the simulation of pulpal fluid flow, and standardizes the lesions depth and bacterial contamination. A brief overview of the experimental steps is given in Fig. 1.

## Results

**Figure 2:**
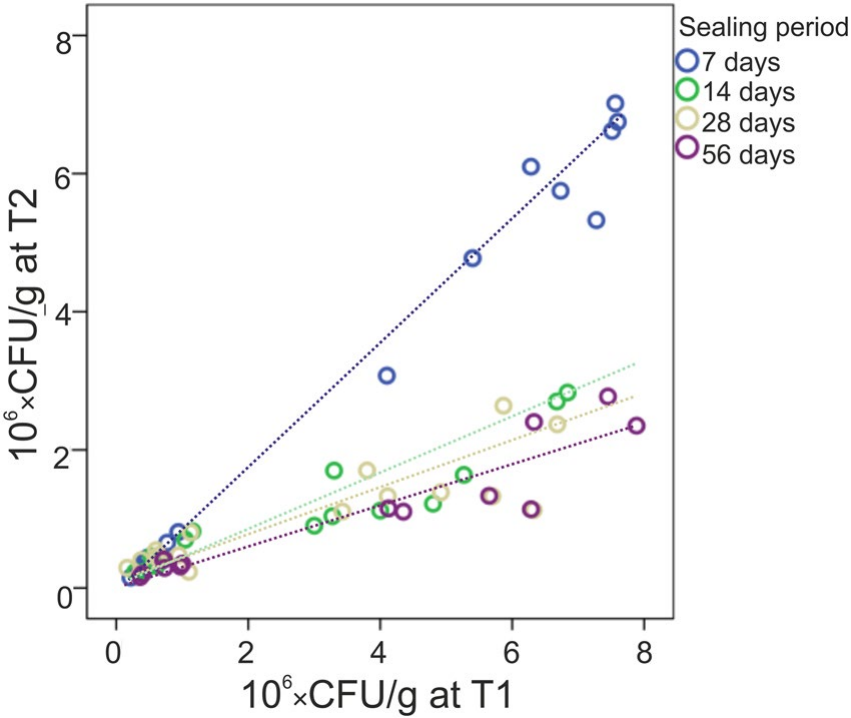
Bacterial numbers of *Lactobacillus rhamnosus* before (T1) and after (T2) sealing in colony-forming units per g dentin (CFU/g). Different sealing periods are indicated by different colors. Longer sealing periods led to lower bacterial numbers at T2, with a linear association between bacterial numbers at T1 and T2 regardless of the sealing period.

**Figure 3:**
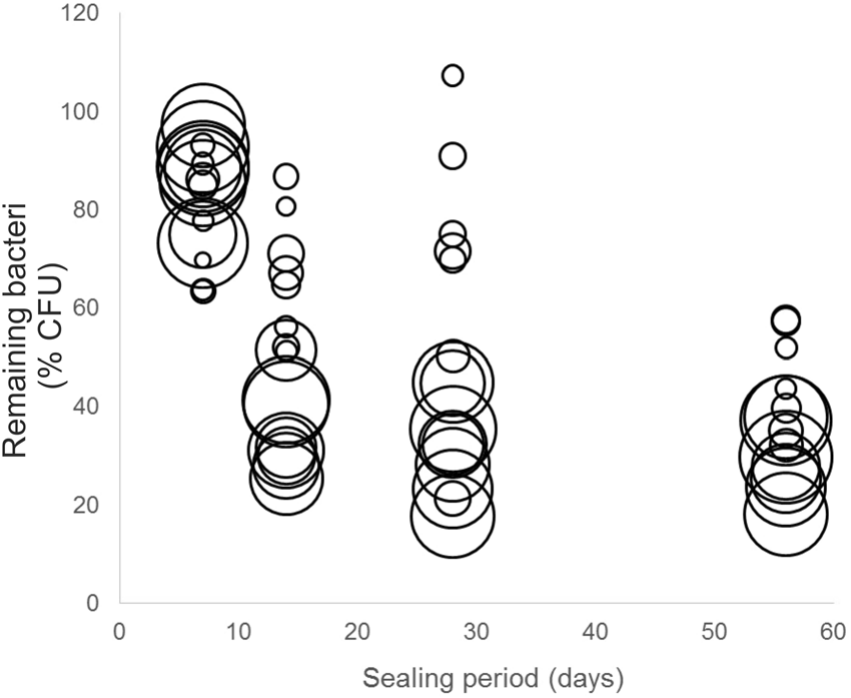
The remaining number of *Lactobacillus rhamnosus* (as colony-forming units per g dentin, CFU/mg, relative to the baseline bacterial load in %) after different sealing periods. The size of each circle indicates the baseline bacterial load (in CFU/g) of the lesion (the larger the circle, the higher the initial load). The reduction followed a first-order kinetics, i.e. it was highest early during sealing in absolute terms, while the relative reduction was near-constant in relative terms (e.g. 23% reduction after 7 days to 77%, which was again reduced by 23% to 59% after 14 days, etc.). In lesions which initially contained more bacteria, the remaining % was lower after sealing (especially after 14 days sealing period, as indicated by larger circles showing the lower remaining %).

We first assessed the survival kinetics of sealed bacteria. For LR, a 2 and 10 days bacterial biofilm cultivation on the dentin discs led to contaminated lesions containing a median (25^th^/75^th^ percentiles) of 0.6 (0.4/0.9) and 5.7 (5.1/6.7) × 10^6^ CFU/g, respectively. The remaining number of LR after sealing was found to be significantly affected by the initial bacterial load (β = 0.53 [95% CI: 0.43/0.62] × 10^6^ CFU/g, p < 0.001) i.e. the remaining number of LR increased by 0.53 × 10^6^ CFU/g per 10^6^ CFU/g being present initially. Similarly, the remaining number of LR decreased with the sealing period (β = −0.03 [95% CI: −0.04/−0.02] × 10^6^ CFU/g, p < 0.001), as depicted in Fig. 2. The relative reduction (in % of CFU/g) of LR followed a first order kinetics (Fig. 3) with R² = 0.31 (p < 0.001), i.e. the relative reduction was largely constant with time, while in absolute terms, the large share of the reduction occurred early during sealing. Moreover, as can also be seen in Fig. 3, lesions with a higher bacterial load showed a larger relative bacterial reduction (i.e. a lower % of remaining bacteria). For SS, the 4 and 9 days bacterial cultivation led to lesions containing 0.5 (0.4/1.4) and 0.7 (0.5/2.1) × 10^6^ CFU/g, respectively. After 7 days of sealing, no viable SS remained regardless of the initial bacterial load.

**Figure 4:**
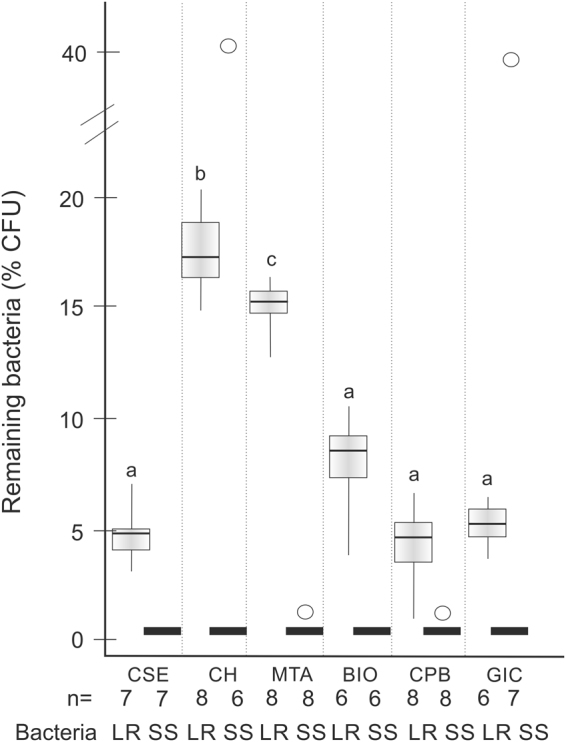
Survival of *Lactobacillus rhamnosus* (LR, light grey, left sub-column, after 2 weeks sealing) and *Streptococcus sobrinus* (SS, black lines or rings, right sub-column, 1 weeks sealing). The remaining colony-forming units per g dentin (CFU/mg) relative to the baseline bacterial load in % is given. Box and line: Interquartile range and median, whiskers: minimum/maximum, rings: outliers, n: sample size. Bacteria were sealed beneath composite placed using Clearfil SE Bond (CSE), lined with calcium hydroxide (CH), mineral trioxide aggregate (MTA) or Biodentine (BIO), placed using Clearfil ProtectBond (CPB) or sealed using glass ionomer cement (GIC). For LR, significant differences are indicated by different superscript letters (p < 0.05, Mann-Whitney/Bonferroni), i.e. significantly more bacteria survived beneath CH than MTA, and beneath MTA than all other groups. For SS, the median survival was zero regardless of the group, and no significant differences were detected.

We then assessed the effect of different sealing materials on the % of remaining bacteria (Fig. 4). For LR, the initial bacterial load for this experiment was 3.7 (2.8/4.4) × 10^6^ CFU/g. CH showed the highest % of remaining LR, followed by MTA (p < 0.001/Mann-Whitney). There was no significant difference between the other materials. For SS, the initial bacterial load for this experiment was 0.7 (0.4/1.1) × 10^6^ CFU/g. None of the materials had a significant different impact on the % of remaining SS. When pooling the results from both bacteria, CH showed the lowest reduction (highest % remaining bacteria), followed by MTA, followed by Biodentine. All other materials (GIC, direct adhesive restoration using Clearfil SE Bond or Clearfil ProtectBond) did not differ in their % of remaining bacteria.

## Discussion

In the present study, we found that the survival of sealed bacteria was strain-specific; LR showed significantly higher % of remaining bacteria than SS after sealing. We thus refute our first hypothesis. Moreover, for LR, longer sealing periods were significantly associated with lower numbers of remaining bacteria, we thus accept our second hypothesis for this strain. We also found that, for LR, the absolute bacterial reduction was largest early during sealing, while the relative reduction was largely constant with time; i.e. the survival followed a first-order kinetics, which has been found for other starving bacterial cultures, especially when the initial bacterial density is high^12^. The underlying mechanism involves a regulatory circle of stress response genes being activated during starvation, which restricts cell growth and substrate consumption until a new equilibrium of nutrition and consumption is reached^12^.

Based on our results, extended sealing periods convey only limited benefits as to the bacterial reduction within the lesion (for LR, the absolute reduction beyond week 2 was limited, while for SS, no viable bacteria were detectable even after one week). This finding, however, should not be readily translated into the clinical setting: First, the bacterial spectrum differs in natural compared with our artificial lesions. The natural bacterial complex is likely to survive longer beneath a sealant compared with our experimental flora^13,14^, as cross-nutrition and bacterial selection processes are likely “optimized” in natural lesions. Second, the tubular patency is usually reduced beneath carious lesions due to pulpal reactions^15^, especially compared with the third molars used in the present study. This could decrease the potential for pulpal bacterial nutrition and accelerate the bacterial inactivation^16,17^. Clinically, longer sealing periods might additionally allow the induction of reactionary dentin, which reduces the risk of pulp exposure when re-entering as part of the stepwise tissue removal^11^.

We also found that leaving more LR beneath a restoration led to significantly higher bacterial numbers remaining after the sealing period, with a near-linear association between both parameters regardless of the sealing period. This is in line with our third hypothesis for this strain. We found that the relative reduction was larger in heavily compared with minimally contaminated dentin, the latter showing only limited or no bacterial reduction at all. Such kinetics have been reported before, as the depletion of nutrients is only slowly escalating in low density cultures (leading to a biphasic survival kinetics), but not high density cultures^12^. Moreover, certain bacteria might be able to deglycosilate pulp glycoproteins and utilize the liberated sugars, as has been found clinically, too^9^. Based on such findings, it is unlikely to render sealed carious lesions completely free of viable bacteria even after longer sealing periods, and one can expect bacteria to regrow to a certain extend beneath sealants or restorations^9,18,19^. The impact of any kind of surviving bacteria on the pulp remains unclear^20^. We are currently adapting our dual-chamber system to accommodate pulpal cell cultures.

In contrast to LR, for SS no or only minimal amounts of cultivable bacteria remained regardless of the initial load and the sealing period. This might have a number of reasons. First, SS may have been less resistant against starvation conditions than LR, as indicated by clinical data^5,21^. Second, SS may have a lower capacity of adapting its metabolic activity, as described above. Third, SS seems to have a limited ability to invade dentinal tubules compared to LR^15,22^. This observation may be due to the strain-specific differences in cell aggregation and cell adhesion, which among other factors depend on the composition of the extracellular matrix produced by the bacteria. The extracellular matrix of SS is formed by homo-exopolysaccharides, mainly mutan, which results in a strong binding to surfaces and between bacterial cells^23–25^. In contrast, LR uses hetero-exopolysaccharides in order to build up a matrix^23,24^. LR is also able to degrade the exopolysaccharides with the help of glycohydrolases during prolonged fermentation^26^. This could allow easier transformation of the biofilm and, generally, lead to a less stable biofilm formation. This may allow LR to enter the dentinal tubules more easily, with subsequently better access to nutrients in the pulpal fluid and better protection against sealing procedures (etching, rinsing) and sealing materials.

We found significant differences between different restorative treatment protocols with regards to the numbers of surviving LR, and thus accept our last hypothesis for this strain. While a number of studies have argued for an antibacterial lining or cavity pretreatment^21,27–30^, our study yields more nuanced results, with CH and MTA lining resulting in more surviving bacteria than Biodentine lining or no lining (i.e. directly restoring the lesion using bonded composite or glass ionomer cement). The limited antibacterial effects of CH have been shown before^31,32^, which raises doubts as to the suitability of this widely used material for treating deep and contaminated dentin. The possible advantages of Biodentine, a calcium silicate cement, over CH have also been demonstrated^33^. Our findings for LR are in line with clinical evidence, too. Such evidence has shown that restoring the lesions without a lining might be clinically beneficial^34^. Given the magnitude of bacterial reduction beneath most materials (around or above 95% in the no lining groups), it is clear that any additionally claimed benefits of specific materials (Clearfil ProtectBond being antibacterial due to its MDPB-component, or glass ionomer cement due to its fluoride release) might be of limited relevance. Clinical studies are needed to confirm these findings. For SS, in contrast, the % of remaining bacteria was near or at zero regardless of the used sealing material, which is due to the high antibacterial effect of sealing itself for this strain. Hence, specific antibacterial materials will not be useful in experiment with SS.

The present study has a number of limitations. First and as discussed, only two bacterial strains were used, while the clinical flora would be more complex, with a vast number of (metabolic) interactions possibly allowing a better adaption to the changed environmental conditions^35^. Second, we assessed standardized lesions, with defined residual dentin thickness, pulpal pressure and fluid composition. Future studies should strive to vary these parameters to increase our understanding as to how they impact on bacterial inactivation via sealing. Third, the simulated pulpal fluid flow was very simple and used tubular transport ways. *In situ*, it is likely mediated by odontoblasts, with regulated ex- or transudation of fluid components (glycoproteins, amino acids, likely also carbohydrates) through capillary vessels into the tubules and the dentin^36^. Fourth, our samples had been sterilized using autoclaving prior to the experiment, which might reduce bond strengths of dental adhesives to the dentin^37^. Given that all groups were sterilized identically, we accepted that. Last, our outcome measure was colony-forming units, which display cultivable bacteria. There is some debate as to the true viability of non-cultivable bacteria, which might regrow in case of sufficient cultural conditions being provided^38^. Alternative methods like imaging (using life-dead staining or fluorescence-*in-situ* hybridization) or polymerase chain reaction might be used in addition. Similarly, we only assessed the number of bacteria, not their activity. To yield a deeper understanding of the bacterial reaction to sealing, future studies should consider to assess transcriptomic and metabolomic activity of sealed bacteria^12^.

The strength of this study lies in the employed experimental setup. For the first time, an *in vitro* device was used which allowed concomitant simulation of oral and pulpal fluid flow during bacterial cultivation. The yielded bacterial numbers are in line with what can be expected when residual carious dentin is sealed clinically, which speaks for the validity of the bacterial simulation system^5,19^. The employed cariogenic bacterial strains were justified from our perspective for this study, as both bacteria are regularly found in deep carious lesions; that are those lesions, which are candidates for selective or stepwise removal^5,19,34,39^. The used simulation system is highly versatile and allows to study not only kinetics and material-related effects, but also metabolomic reactions of bacteria to sealing etc.

The present study used a novel dual-chamber device to assess the survival kinetics of sealed dentin bacteria. Based on our findings, the bacterial survival was strain-specific. For SS, sealing reduced all cultivable bacteria regardless of the employed initial bacterial load, sealing material or sealing period. For LR, the survival follows a first-order kinetics with the absolute bacterial reduction being largest in the early sealing period. Sealing larger number of bacteria led to more bacteria surviving. However, only low numbers of sealed bacteria seem to be able to survive long-term. Different materials had a different potency to reduce the number of surviving bacteria. It should be highlighted that the relevance of remaining bacteria for lesion arrest and pulpal vitality needs to be explored, and that clinically, a large number of further aspects (selective reduction of specific strains, pulpal inflammatory status, pulpal cell reaction to restorative materials) are likely to impact on treatment success. With these limitations in mind, the developed simulation system is suitable to assess sealed bacteria *in vitro*.

## Methods

### Sample preparation

Two hundred and eighty extracted permanent third human molars without any caries lesions or restorations were obtained under informed consent within an ethics-approved protocol (ethics committee of the Charité – Universitätsmedizin Berlin EA4/102/14). All experiments described in the following sections were performed in accordance with relevant guidelines including good laboratory practice, and the regulations of this ethics committee. Both roots and coronal enamel were removed (Band Saw 300 cl; Exakt Apparatebau, Norderstedt, Germany), the specimens embedded using acrylate resin (Technovit 7042, Heraeus Kulzer, Hanau, Germany) in round molds (Ø = 9.5 mm), the pulpal surface flattened to the level of the pulp horns, the dentin discs plan-parallelized to a standardized thickness of 1.5 mm and the surfaces eventually polished (Mikroschleifsystem 400 CS, Exakt). The discs were then trimmed to a round shape with a diameter of 9 mm using a trepan bur (Komet, Lemgo, Germany). The pulpal surface was uncovered using 0.5 M EDTA (pH = 5.0) for 2 min to remove the smear layer and open the dentinal tubules^40^. The latter was controlled in a pilot-study on five discs using scanning electron microscopy^41^. No attempts of removing the smear layer on the coronal surface were made. The discs were then sterilized at 121 °C, 2.1 bar for 20 min.

### Dual-chamber device

The dual-chamber pulpal simulation system was custom-made and constructed from stainless steel. The two chambers simulated the two relevant compartments; the oral cavity and the pulpal cavity. The two chambers were separated by the dentin discs. These were mounted in the device using silicone hoses (inner Ø = 9 mm, VWR, Leuven, Belgium) which served as adapter to a telescopic pipe. The tight connection between discs, hose and pipe had been checked using methylene blue and bacterial broth leakage tests in pilot studies. The coronal surface of each disc was directed toward the outside of the telescopic pipe, i.e. the simulated mouth chamber, which had three fluid entrances and one waste fluid exit. The pulpal surface of each disc was connected to the simulated pulpal chamber via the hose and the pipe (Fig. 1).

### Induction of bacterially contaminated lesions

To induced bacterially contaminated lesions, an outer ring of each coronal surface was covered with nail-varnish (Rival de Loop, Rossmann, Hannover, Germany), leaving a round window (Ø = 4 mm) uncovered for lesion induction. Coronal surfaces were now submitted to a previously validated protocol: First, surfaces were pre-demineralized using either acetic acid for 7 days (for LR), or acetic acid for 2 to 10 days (for SS)^42^. Bacterial biofilms of LR (DSM 20021 DSMZ, Braunschweig, Germany) and SS (DSM 20742, DSMZ, Braunschweig, Germany) were then cultured on these demineralized surfaces, allowing bacteria to invade the dentin. To do so, a computer-controlled continuous-culture biofilm model was used^43^, with overnight cultures of bacteria in deMan-Rogosa-Sharpe (MRS) broth (Carl Roth, Germany) for LR and brain-heart-infusion (BHI) broth (Carl Roth, Germany) for SS being incubated on the coronal surfaces within the mouth chamber at 100% humidity at 37 °C for 15 min. Afterwards, 250 ml MRS or BHI (for LR or SS), supplemented with 2% sucrose, was provided over 10 min using peristaltic multi-canal pumps (8152 Standard, MCP, Glattbrugg, Germany). Biofilms were allowed to rest in 100% humid atmosphere for 24 hours, after which the cycle was repeated.

For the first experiment (bacterial survival kinetics), bacteria were cultured for 2 and 10 days for LR and 4 and 9 days for SS, respectively, to induce differently contaminated lesions. For the second experiment, incubation time was 10 d for LR and 15 d for SS. Note that for LR, samples were mounted within the chamber prior to biofilm induction, as this was easier to handle during the subsequent steps. For SS, samples were mounted after the restoration (see below), as cultivation of SS biofilms was more successful when samples were not hanging upwards (i.e. mounted).

### Restoration

The coronal surface was now restored as would be done in a clinical setting, with the restoration sealing the bacterially contaminated lesion. To do so, we first removed the biofilm using a sterile swap. In the first experiment, the restoration was then performed as follows: A self-etch adhesive (iBond, Heraeus Kulzer, Hanau, Germany) was applied for 15 s, carefully blown dry and light-cured for 20 s with an LED-light-curing with an intensity of 950 mW/cm^2^ (Smartlite, Dentsply Detrey, Konstanz, Germany), followed by the placement of a flowable composite (Tetric EvoFlow, Ivoclar Vivadent, Schaan, Liechtenstein) and another 20 s of light-curing as described. In the second experiment, a number of restoration materials were tested for their effects on bacterial survival:

1. Direct restoration using another self-etch adhesive (Clearfil SE Bond, Kuraray, Tokyo, Japan), without any liner, followed by the placement of the flowable composite (Tetric EvoFlow) and 20 s of light-curing.
2. Placement of a calcium hydroxide (CH) liner (Dycal, Kerr, Scafati Salerno, Italy) in a standardized thickness of 1.0 ± 0.2 mm onto the lesion (leaving the sound dentin ring free of any lining material), with subsequent bonding procedure in sound areas using Clearfil SE Bond as described, and coverage with Tetric EvoFlow.
3. Placement of mineral trioxide aggregate (MTA) liner (ProRoot MTA, Dentsply DeTrey) and restoration as described.
4. Placement of Biodentine (Septodont, Niederkassel, Germany) and restoration as described.
5. Placement of an antibacterial self-etch adhesive (Clearfil ProtectBond, Kuraray) and restoration as described.
6. Glass ionomer cement restoration (Equia Forte, GC, Tokyo, Japan) without any conditioning of the surface.

### Simulation phase

After restoring the coronal surfaces, the mouth chamber was flooded with artificial saliva (defined mucine medium, DMM) to simulate the oral milieu^44^. DMM was renewed weekly. The pulpal chamber was flooded with simulated pulpal fluid (SPF), containing 30 mmol/l Hepes buffer, 0.93 mmol/l CaO, 0.60 mmol MgO, 77.6 mmol/l, 1.1 mmol/l H_3_PO_4_ and 20% albumin^45^. A constant pulpal pressure of 1.96 kPa^46,47^ and an SPF circulation was generated as follows: The pulpal chamber consisted of a two-way fluid transport system. The SPF reservoir was stored 20 cm above dentin disc, and SPF was passively flooded onto the pulpal surface of the dentin discs via telescopic down-pipes (inner Ø = 10 mm), generating 1.96 kPa hydrostatic pressure. SPF was then slowly pumped back into the SPF reservoir via inner up-pipes (inner Ø = 2 mm), which were placed directly above the dentin discs to ensure SPF circulation (Fig. 1). Equal fluid flows through all elements of the device had been tested in a pilot study. Note that for the second experiment with SS, the oral chamber was exposed to SPF for reasons of simplification.

Sealing periods were varied between 7 and 56 days for the first experiment. The sealing period was 7 days (SS) and 14 days (LR) for the second experiment (as sufficient bacterial reductions had been detected after that period in experiment 1).

### Analysis

Our outcome parameter was the number of remaining bacteria after sealing, measured as colony-forming units per g dentin [CFU/g]. As we aimed to assess bacterial reduction, we sampled carious dentin prior to the sealing period (T1) in one half of the lesion, and after the sealing period (T2) in the other half of the lesion. Sampling was performed using a sterile hand excavator, and the net weight of the sampled dentin assessed (Analytical Plus, Ohaus, Nänikon, Switzerland). The dentin was then transferred to 0.9% sodium chloride, the medium serially diluted from 10^−1^–10^−6^ and plated onto MRS agar (Oxoid, Germany) for LR and onto COLS + agar (Oxoid, Germany) for SS regarding enumeration of CFU/g after 48 h at 37 °C. To evaluate for the presence of contaminating species, microscopic assessment of 5 random colonies from each plate was performed. It should be noted that this method is unable to confirm the presence of the specific employed strain.

Statistical analysis was performed using SPSS 20 (IBM, Armonk, USA). Data was controlled for normal distribution using Shapiro-Wilk-test. Different groups were compared using Mann-Whitney-U test, with Bonferroni correction for alpha-inflation. Ordinary least square regression analysis was performed to assess the impact of different independent variables on our outcome parameter. The level of significance was set at p < 0.05 and tests were performed two-sided.

All data generated or analyzed during this study are included in this published article.

### Data availability statement

All data generated or analysed during this study are included in this published article.

### Ethical approval

As described, all experimental protocols were approved the Charité ethics committee. Our methods were carried out in accordance with the relevant guidelines and regulations. The use of human tooth tissue was possible after obtaining informed consent from all participants and/or their legal guardian/s.
